# National and Subnational Incidence, Mortality, and Years of Life Lost Due to Breast Cancer in Iran: Trends and Age-Period-Cohort Analysis Since 1990

**DOI:** 10.3389/fonc.2021.561376

**Published:** 2021-03-25

**Authors:** Bahar Ataeinia, Sahar Saeedi Moghaddam, Mahsima Shabani, Kimiya Gohari, Ali Sheidaei, Nazila Rezaei, Shohreh Naderimagham, Erfan Ghasemi, Mahtab Rouhifard Khalilabad, Shahin Roshani, Yosef Farzi, Farshad Farzadfar

**Affiliations:** ^1^Non-Communicable Diseases Research Center, Endocrinology and Metabolism Population Sciences Institute, Tehran University of Medical Sciences, Tehran, Iran; ^2^Center for Precision Imaging, Department of Radiology, Massachusetts General Hospital, Boston, MA, United States; ^3^International Hematology/Oncology of Pediatrics Experts (IHOPE), Universal Scientific Education and Research Network (USERN), Tehran, Iran; ^4^Department of Biostatistics, Faculty of Medical Sciences, Tarbiat Modares University, Tehran, Iran; ^5^Department of Epidemiology and Biostatistics, School of Public Health, Tehran University of Medical Sciences, Tehran, Iran; ^6^Endocrinology and Metabolism Research Center, Endocrinology and Metabolism Clinical Sciences Institute, Tehran University of Medical Sciences, Tehran, Iran

**Keywords:** age-period-cohort model, breast cancer, incidence, mortality, time trend

## Abstract

Breast cancer is the most common cancer among women, causing considerable burden and mortality. Demographic and lifestyle transitions in low and low-middle income countries have given rise to its increased incidence. The successful management of cancer relies on evidence-based policies taking into account national epidemiologic settings. We aimed to report the national and subnational trends of breast cancer incidence, mortality, years of life lost (YLL) and mortality to incidence ratio (MIR) since 1990. As part of the National and Subnational Burden of Diseases project, we estimated incidence, mortality and YLL of breast cancer by sex, age, province, and year using a two-stage spatio-temporal model, based on the primary dataset of national cancer and death registry. MIR was calculated as a quality of care indicator. Age-period-cohort analysis was used to distinguish the effects of these three collinear factors. A significant threefold increase in age-specific incidence at national and subnational levels along with a twofold extension of provincial disparity was observed. Although mortality has slightly decreased since 2000, a positive mortality annual percent change was detected in patients aged 25–34 years, leading to raised YLLs. A significant declining pattern of MIR and lower provincial MIR disparity was observed. We observed a secular increase of breast cancer incidence. Further evaluation of risk factors and developing national screening policies is recommended. A descending pattern of mortality, YLL and MIR at national and subnational levels reflects improved quality of care, even though mortality among younger age groups should be specifically addressed.

## Introduction

Cancer is a major health dilemma worldwide, responsible for more than 70 million deaths globally since 2010 and a barrier to increasing life expectancy and premature mortality decline in both developed and developing countries (1, 2). Breast cancer is the most prevalent malignancy in women and the second most common malignancy overall, with more than 2 million new cases and 627000 deaths estimated globally in 2018 (1). Breast cancer has been the fifth leading cause of cancer-related deaths worldwide since 2015 (1, 3, 4). In the Eastern Mediterranean Region (EMR), including Iran, breast cancer has had the highest incidence and mortality compared to other cancers among women (5–7).

Although countries with high Socio-Demographic Index (SDI) still have higher breast cancer incidences, demographic and lifestyle transitions in low and low-middle SDI countries has given rise to increasing cancer incidence rates (1). In these regions, limited resources and lack of well-organized health policies can lead to inefficient diagnoses and treatment strategies, causing increased burden and mortality (6, 8, 9). The successful prevention and control of cancer relies on evidence-based policies that take into account national epidemiologic settings and the pattern of local distribution of associated risk factors. For instance, the reported mean age of breast cancer diagnosis in Iran in a number of studies was 10 years lower than the average of high SDI countries (10–13). In this scenario, policy makers would have to design special screening strategies in addition to the established international strategies. On the other hand, even within populations of the same ethnicity and similar SDI levels, there are confounding issues. Mammography, the routine breast cancer screening modality, does not have ideal sensitivity when used in younger ages, while creating patient anxiety over diagnosis and radiation exposure. The latter side effects can cause more harm than good (14). Other proper modalities for the younger population, including Magnetic Resonance Imaging (MRI), still need more studies on efficacy evaluation and might not be widely available or covered by insurance (15). Such issues highlight the need for fundamental alterations in the health system and a strict resource allocation in resource-limited regions. This example of screening strategies emphasizes the significance of precise epidemiologic studies and well-organized Population-Based Cancer Registries (PBCRs) at national and subnational levels as the initial steps toward establishing appropriate policies (6, 16, 17). Although the Global Burden of Diseases (GBD) and global cancer incidence, mortality and prevalence (GLOBOCAN) studies provide valuable estimates on global and regional cancer epidemiology, they are not substitutes for PBCRs, especially at subnational levels (1, 16).

Even though a number of studies have reported breast cancer incidence and mortality based on local cancer registries or the National Cancer Registry (NCR) (11, 18–20), there is a considerable paucity of literature on the precise updated data of incidence, mortality and burden of breast cancer in Iran, especially at subnational levels. To the best of our knowledge, limitations of the NCR, such as incompleteness and misclassification, have not been addressed and systematically controlled in previous studies. Therefore, we aimed to report female breast cancer incidence, mortality and Years of Life Lost (YLL) during more than 2 decades at national and subnational levels along with an age-period-cohort analysis. In addition, we have reported Mortality-Incidence Ratio (MIR) as an indicator of cancer management outcome and quality of care.

## Material and Methods

This study was part of the Cancer subdivision of the National and Subnational Burden of Diseases (NASBOD) project that provides estimates on incidence, mortality and burden of different diseases as well as attributed burden to risk factors in Iran over a 26-year period at national and subnational levels (21). We estimated breast cancer incidence, mortality, YLL and MIR for a 26-year period between 1990 and 2016 at national and provincial levels in Iran. Comprehensive details of methodology are presented in protocol papers of mortality and incidence (22). Results of the present study can be accessed online using data visualization tools at www.vizit.report website.

### Data Source

We utilized the Iranian databases of the Cancer Registration System (CRS) (from 2000 to 2010, excluding 2006) and Death Registration System (DRS) (from 1995 to 2010, excluding 2005). Also, data from cemeteries in Tehran (from 1995 to 2010) and Esfahan (from 2007 to 2010) were added to the DRS data set; while information of other cemeteries in the country were registered in the national DRS. All registration systems encounter incompleteness. In order to estimate incompleteness in the CRS, the Social Security Organization Cancer Registry (SSOCR) data was used, since all cancer patients should be registered in the SSOCR database prior to receiving cancer medications. In a parallel study, Mohammadi et al. estimated the levels and trends of child and adult mortality and their results were used to estimate incompleteness of the DRS (23–27).

Several covariates including wealth index, average successful years of schooling, and urbanization rates were extracted from the Household Income and Expenditure Survey and Population and Housing Censuses datasets to be used in the imputation of missing values in the CRS and DRS.

### Variable Definition

Cancer type, year of initial diagnosis, patients’ demographics (age, sex) and province of residence were extracted from registration systems. In case of missing sex for registered patients, other available identification characteristics were used to impute the missing data points. Iran consists of 31 provinces since 2011 (according to the administrative divisions of Iran’s provinces). Due to alterations in the number of provinces in the study period, we used district names and postal codes for provincial remodeling based on 2011’s subdivisions (there were no changes in the number of provinces after 2011) and to prevent possible data misalignments.

In the CRS, cancer type was registered according to the International Classification of Diseases for Oncology (ICD-O), and the 10^th^ revision of the International Classification of Diseases (ICD-10). These codes were then mapped to 18 main types and 70 subtypes of cancers by a team of physician experts. In the DRS, 567 ICD-10 codes including the underlying causes of death and garbage codes were mapped to 165 GBD causes of death.

### Statistical Analysis

Statistical analysis was conducted using R statistical software and STATA version 11.0 (STATA Corp., College, Station, TX, USA). After using available identification characteristics for the initial step of imputation, Amelia package was utilized to impute the remaining missing values (28). A text mining algorithm in Python software was used to detect all types of duplicate entries.

All-cause incidence and mortality rates were modeled in two phases. First, we used a random effect model and in the next step, we re-modelled the residuals by an “age-spatio-temporal” model (26). We applied weights to take into consideration the neighboring provinces for location and applied the weight matrix for age differences among age groups combined with a smoothing hyperparameter. Multinomial logistic regression approach was utilized to model incidence and mortality of cause fractions and applied to all-cause incidence and mortality by age, sex, province, and year.

In order to estimate 95% Uncertainty Intervals (95% UI), 1,000 random normal values from the distribution of the mixed effect model were predicted. After applying the spatio-temporal model to these values 2.5^th^ and 97.5^th^ percentiles were chosen as the lower and upper bounds of UIs, respectively.

YLL was calculated by multiplying the number of deaths by life expectancy of Iranian people at each age, sex, province and year (29). Life expectancy was taken from another part of the NASBOD study which was estimated by Mehdipour et al. (30) at national and subnational levels.

We used decomposition analysis to determine the contribution of change in the age-specific incidence rate, population growth and population aging on the absolute change of new cancer cases between 1990 and 2016. Age and sex structure and age-specific rates of 1990 were applied to the total population of 2016; considered as the first hypothetical data. The difference between new cases in 1990 and the first hypothetical data was attributed to population growth. Upon applying age-specific rates from 1990 to age and sex structure and population in 2016 they were considered as the second hypothetical data. The difference between the second and first hypothetical data was attributed to population aging. Attribution to change in the age-specific incidence rates was considered as the difference between new cases in 2016 and the second hypothetical data (3).

We performed an age-period-cohort modeling with the Intrinsic Estimator (IE) method by Yang et al. (31) to decompose the effects of these three collinear factors. The “apc-ie” command in STATA software was utilized for analysis. Data were stratified in 19 birth cohorts starting from 1905-1909, four five-year calendar periods starting from 1990 and 15 five-year age groups starting from 15 years of age.

The exponential regression coefficient from the regression of the natural logarithm (ln) of incidence rate on year was considered as the Annual Percent Change (APC) (32). We computed age-standardized rates by the direct method of standardization in the ‘epitools’ package (33) in R3.0.2 and utilizing the Iranian population in the last year of each study as the reference population.

## Results

At national level, the Age-Standardized Incidence Rate (ASIR) of breast cancer increased by more than three folds from 13 (95% UI: 7.5 to 20.0) cases per 100,000 individuals in 1990 to 44 (36.4 to 52.0) cases per 100,000 individuals in 2016. APC was calculated at 4.8% for incidence rate. An overall eight-fold increase in the number of new cases was observed since 1990, at 1452 (777 to 2,382) new cases and 14,217 (11,764 to 16,801) new cases in 2016 (**Table 1**)

**Table 1:** Number, rate and APC of Incidence, mortality and YLL at national and subnational levels.

Location	Incidence					Death					YLL				
	1990		2016		1990 to 2016	1990		2015		1990 to 2015	1990		2015		1990 to 2015
	All ages (number)	Age standardized (rate per 100,000)	All ages (number)	Age standardized (rate per 100,000)	APC (%)	All ages (number)	Age standardized (rate per 100,000)	All ages (number)	Age standardized (rate per 100,000)	APC (%)	All ages (number)	Age standardized (rate per 100,000)	All ages (number)	Age standardized (rate per 100,000)	APC (%)
National	1452 (777 to 2382)	13 (7.5 to 20)	14217 (11764 to 16801)	44 (36.4 to 52)	4.8	594 (458 to 769)	5.7 (4.4 to 7.4)	1608 (1274 to 2031)	5.1 (4.1 to 6.5)	-0.5	9756 (7502 to 12692)	85.1 (65.6 to 110.4)	47491 (37618 to 59908)	151.1 (119.7 to 190.6)	2.3
Alborz	24 (11 to 44)	11.1 (5.9 to 18.1)	401 (319 to 490)	38.7 (31.3 to 46.7)	4.9	8 (6 to 10)	4.3 (3.4 to 5.6)	101 (80 to 127)	10 (7.9 to 12.6)	3.4	153 (119 to 198)	71.8 (55.8 to 92.3)	2473 (1956 to 3119)	229.8 (181.7 to 289.9)	4.8
Ardebil	23 (11 to 41)	10.8 (5.7 to 17.5)	164 (131 to 200)	32.1 (25.7 to 39.2)	4.3	9 (8 to 12)	4.9 (4 to 6.1)	21 (18 to 25)	4.4 (3.7 to 5.3)	-0.4	164 (131 to 204)	73.5 (59.1 to 91.1)	582 (484 to 695)	117.3 (97.6 to 140.3)	1.9
Bushehr	9 (3 to 21)	6.8 (2.7 to 13.8)	119 (87 to 157)	32.2 (24.4 to 41.5)	6.2	5 (4 to 6)	4.3 (3.4 to 5.3)	21 (17 to 26)	6.1 (4.9 to 7.5)	1.4	84 (67 to 105)	67.7 (54.1 to 84.5)	563 (455 to 692)	152.8 (123.7 to 187.8)	3.3
Chahar Mahall and Bakhtiari	13 (7 to 22)	10.2 (5.9 to 15.4)	106 (85 to 128)	30.2 (24.6 to 36.3)	4.3	5 (4 to 6)	3.9 (3.2 to 4.8)	13 (10 to 15)	3.7 (3.1 to 4.5)	-0.2	83 (68 to 103)	61.6 (50 to 75.7)	336 (277 to 408)	98.6 (81.2 to 119.6)	1.9
East Azarbaijan	92 (51 to 144)	13.4 (7.9 to 19.9)	682 (570 to 797)	40.3 (33.4 to 47.5)	4.3	40 (32 to 49)	6.4 (5.2 to 7.9)	76 (63 to 92)	4.6 (3.8 to 5.6)	-1.3	592 (475 to 739)	83.2 (66.9 to 103.3)	2419 (2001 to 2927)	151.2 (125 to 183)	2.4
Esfahan	98 (52 to 162)	12.4 (6.9 to 19.2)	1213 (1017 to 1413)	53.1 (44.2 to 62.2)	5.8	29 (23 to 37)	3.8 (3 to 4.8)	119 (94 to 149)	5.4 (4.3 to 6.8)	1.4	608 (477 to 775)	73.6 (57.9 to 93.6)	3069 (2441 to 3842)	142.6 (113.4 to 178.5)	2.7
Fars	101 (57 to 157)	14.7 (8.8 to 21.6)	1042 (873 to 1217)	52.5 (44 to 61.2)	5	23 (19 to 29)	3.6 (2.9 to 4.4)	110 (89 to 136)	5.7 (4.6 to 7)	1.9	378 (304 to 470)	54.3 (43.8 to 67.1)	3394 (2740 to 4190)	173.6 (140.2 to 214.2)	4.8
Gilan	48 (26 to 77)	8.8 (4.9 to 13.7)	465 (391 to 540)	35 (28.8 to 41.3)	5.4	20 (16 to 26)	4.1 (3.3 to 5.1)	69 (55 to 85)	5.3 (4.3 to 6.6)	1.1	315 (250 to 394)	57.6 (46 to 72)	1929 (1551 to 2392)	155.2 (124.7 to 192.7)	4
Golestan	15 (7 to 29)	6.4 (3.2 to 10.8)	184 (144 to 228)	27 (21.3 to 32.9)	5.7	9 (8 to 11)	4.6 (3.8 to 5.7)	35 (28 to 42)	5.2 (4.3 to 6.3)	0.5	149 (122 to 183)	63.1 (51.5 to 77.2)	898 (733 to 1095)	126.9 (103.6 to 154.5)	2.8
Hamadan	28 (15 to 47)	8.7 (4.8 to 13.4)	289 (240 to 338)	36.2 (30 to 42.7)	5.7	11 (9 to 13)	3.7 (3.1 to 4.5)	35 (29 to 41)	4.5 (3.8 to 5.4)	0.8	200 (165 to 244)	58.3 (48.1 to 70.9)	1034 (866 to 1235)	140.9 (118 to 168.4)	3.6
Hormozgan	7 (2 to 19)	3.7 (1 to 9.5)	116 (77 to 169)	20.9 (14.5 to 28.9)	6.8	5 (4 to 6)	2.5 (2 to 3.2)	23 (19 to 29)	4.5 (3.6 to 5.7)	2.4	73 (57 to 93)	42.9 (33.5 to 54.9)	712 (566 to 893)	130.2 (103.5 to 162.9)	4.5
Ilam	6 (3 to 10)	7.4 (3.8 to 11.9)	58 (46 to 71)	27.3 (22.1 to 33)	5.1	2 (2 to 3)	3.5 (2.8 to 4.5)	10 (8 to 12)	4.8 (3.8 to 5.9)	1.2	57 (45 to 72)	72.8 (57.5 to 91.7)	294 (235 to 364)	132.1 (105.9 to 163.8)	2.4
Kerman	45 (25 to 70)	12.7 (7.6 to 18.6)	385 (311 to 459)	33.1 (27.1 to 39.1)	3.7	14 (11 to 17)	4.2 (3.5 to 5.1)	41 (34 to 50)	3.7 (3 to 4.5)	-0.5	232 (192 to 281)	63.6 (52.6 to 76.9)	1291 (1051 to 1580)	113.5 (92.5 to 138.7)	2.3
Kermanshah	34 (18 to 56)	11.6 (6.7 to 17.4)	341 (284 to 399)	40.3 (33.5 to 47.3)	4.9	9 (8 to 11)	3.5 (2.8 to 4.2)	44 (36 to 53)	5.4 (4.5 to 6.5)	1.8	182 (149 to 223)	57.2 (46.9 to 69.6)	1322 (1095 to 1600)	161.2 (133.4 to 194.9)	4.2
Khuzestan	97 (57 to 145)	17.3 (10.9 to 24.6)	1003 (848 to 1161)	63.1 (54 to 72.2)	5.1	17 (14 to 21)	3.2 (2.6 to 3.9)	89 (73 to 109)	5.7 (4.7 to 6.9)	2.4	279 (226 to 345)	50.8 (41.4 to 62.5)	2697 (2212 to 3283)	160.5 (131.7 to 195.2)	4.7
Kohgiluyeh and Buyer Ahmad	8 (4 to 14)	9.7 (5.1 to 15.2)	79 (61 to 97)	32.6 (26 to 39.5)	4.8	2 (2 to 3)	3.4 (2.7 to 4.4)	8 (6 to 9)	3.3 (2.6 to 4.1)	-0.2	43 (33 to 55)	55.3 (42.8 to 71.1)	232 (186 to 288)	94.3 (75.6 to 117.1)	2.2
Kordestan	35 (16 to 63)	14.9 (7.6 to 24.5)	220 (169 to 276)	36.4 (28.3 to 45.3)	3.5	10 (8 to 13)	4.7 (3.7 to 6)	18 (15 to 22)	3.1 (2.5 to 3.7)	-1.7	169 (133 to 215)	72.4 (57.1 to 91.7)	603 (496 to 731)	100.4 (82.7 to 121.6)	1.3
Lorestan	40 (20 to 68)	15.4 (8.5 to 23.7)	251 (201 to 308)	35.3 (28.3 to 43)	3.2	10 (8 to 13)	4.3 (3.4 to 5.3)	23 (19 to 28)	3.4 (2.8 to 4)	-0.9	181 (145 to 227)	65.2 (52.3 to 81.3)	748 (621 to 899)	107.3 (89.1 to 128.9)	2
Markazi	18 (8 to 33)	6.7 (3.1 to 11.6)	281 (234 to 331)	42.5 (34.9 to 50.4)	7.3	8 (6 to 10)	3 (2.4 to 3.7)	36 (29 to 43)	5.5 (4.5 to 6.6)	2.4	155 (125 to 192)	58.1 (47 to 71.9)	1021 (841 to 1236)	166.1 (136.8 to 201.3)	4.3
Mazandaran	54 (28 to 89)	9.9 (5.5 to 15.1)	453 (370 to 537)	29.8 (24 to 35.7)	4.3	22 (18 to 28)	4.4 (3.5 to 5.6)	71 (56 to 90)	4.8 (3.7 to 6.1)	0.3	345 (272 to 436)	62.4 (49.3 to 78.7)	2033 (1589 to 2601)	138.3 (107.9 to 177)	3.2
North Khorasan	11 (5 to 19)	8.3 (4.3 to 13.7)	73 (55 to 93)	21.1 (16.1 to 26.9)	3.6	6 (4 to 7)	5.3 (4.2 to 6.7)	13 (11 to 16)	4 (3.2 to 4.9)	-1.2	71 (57 to 90)	55.1 (43.9 to 69.2)	379 (305 to 471)	111 (89.2 to 137.7)	2.8
Qazvin	11 (5 to 22)	6.4 (3 to 11.2)	166 (133 to 202)	32.7 (26.2 to 39.6)	6.5	8 (7 to 10)	4.8 (3.9 to 5.8)	27 (23 to 33)	5.5 (4.5 to 6.6)	0.5	143 (116 to 176)	76 (61.8 to 93.1)	832 (689 to 1005)	167.7 (138.8 to 202.4)	3.2
Qom	14 (7 to 25)	9.5 (5.1 to 15.8)	137 (107 to 170)	32 (25.7 to 39.2)	4.8	9 (7 to 12)	6.6 (5 to 8.6)	23 (18 to 30)	5.5 (4.3 to 7.1)	-0.7	143 (109 to 189)	102.9 (78.3 to 135.3)	664 (517 to 847)	149.2 (116 to 190.7)	1.5
Razavi Khorasan	115 (64 to 178)	12.8 (7.6 to 18.8)	1164 (977 to 1351)	47.2 (39.7 to 54.7)	5.1	51 (41 to 64)	6.2 (5 to 7.7)	86 (69 to 107)	3.6 (2.9 to 4.4)	-2.2	788 (629 to 990)	83.3 (66.8 to 104.3)	2707 (2170 to 3368)	112.8 (90.5 to 140.2)	1.2
Semnan	10 (5 to 17)	9.1 (4.8 to 14.7)	130 (108 to 154)	45 (37 to 53.4)	6.3	5 (4 to 7)	4.8 (3.7 to 6.2)	19 (15 to 24)	6.8 (5.4 to 8.5)	1.4	79 (62 to 102)	69.1 (53.6 to 88.8)	527 (417 to 661)	195 (154.3 to 244.6)	4.2
Sistan and Baluchestan	16 (6 to 31)	6.6 (2.9 to 11.5)	121 (85 to 165)	17.3 (13 to 22.2)	3.8	8 (6 to 11)	4.3 (3.3 to 5.6)	18 (14 to 22)	2.6 (2.1 to 3.3)	-2	137 (105 to 181)	60 (46 to 78.8)	623 (490 to 791)	86.4 (68.3 to 109.3)	1.5
South Khorasan	8 (4 to 14)	5.8 (3 to 9.7)	57 (45 to 71)	20.8 (16 to 26)	5	5 (4 to 6)	3.4 (2.7 to 4.3)	13 (10 to 16)	4.8 (3.9 to 5.9)	1.4	66 (53 to 84)	45.7 (36.2 to 57.7)	375 (302 to 464)	150.1 (120.9 to 185.9)	4.9
Tehran	371 (213 to 576)	21.5 (13.3 to 31.4)	3709 (3145 to 4289)	63.7 (53.7 to 74.1)	4.3	204 (147 to 283)	12.4 (8.9 to 17.1)	374 (275 to 509)	6.5 (4.8 to 8.9)	-2.5	3135 (2253 to 4334)	170.1 (122.4 to 234.9)	11632 (8567 to 15754)	204 (150.3 to 276.3)	0.7
West Azarbaijan	54 (26 to 98)	12.3 (6.6 to 20.3)	442 (355 to 539)	34.4 (27.5 to 41.8)	4	21 (17 to 26)	6.1 (4.9 to 7.5)	36 (30 to 43)	2.9 (2.4 to 3.5)	-2.9	250 (200 to 314)	56.6 (45.4 to 70.8)	1267 (1046 to 1526)	102.3 (84.4 to 123.1)	2.4
Yazd	27 (16 to 39)	16 (9.9 to 22.9)	301 (258 to 344)	59.3 (50.6 to 68.1)	5.2	8 (7 to 10)	4.8 (3.9 to 5.9)	28 (22 to 34)	6.1 (4.9 to 7.5)	1	128 (103 to 159)	76.2 (61.5 to 94.5)	772 (623 to 952)	179.8 (145.1 to 221.6)	3.5
Zanjan	21 (6 to 53)	12 (4.2 to 27.1)	62 (37 to 104)	14.6 (8.7 to 24.3)	0.8	9 (7 to 12)	6.1 (4.8 to 7.8)	10 (7 to 13)	2.4 (1.8 to 3.1)	-3.7	140 (110 to 178)	78.2 (61.3 to 99.6)	311 (235 to 409)	75.4 (56.9 to 99)	-0.1

. We conducted a decomposition analysis to determine the magnitude of effect for population growth and age structure change on the increased incidence. From the overall 879% increase in new cases, 122.4% of change was attributable to population growth, 67.5% was attributable to age structure change and a considerable increase of 689.2% was caused by an increase in the age-specific incidence rates of breast cancer (**Table S1**).

**Figure 1:**
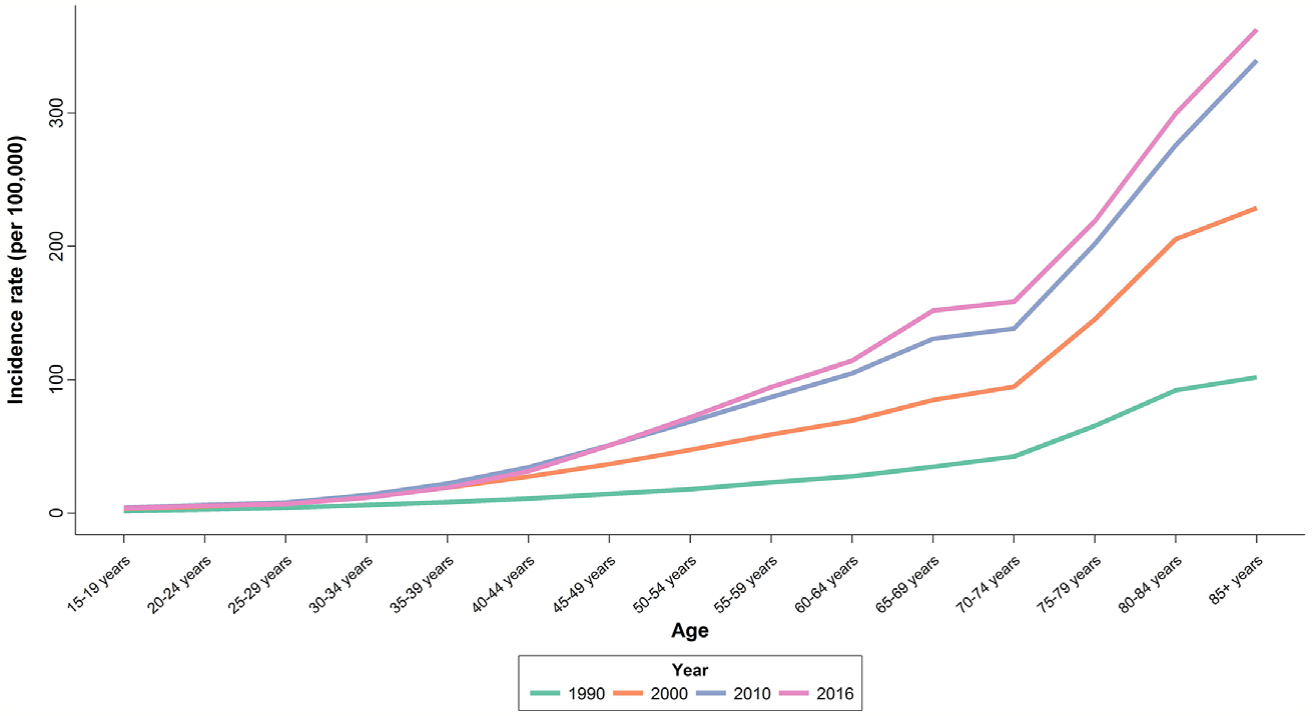
National age trend of incidence rate in 1990, 2000, 2010, and 2016.

**Figure 2:**
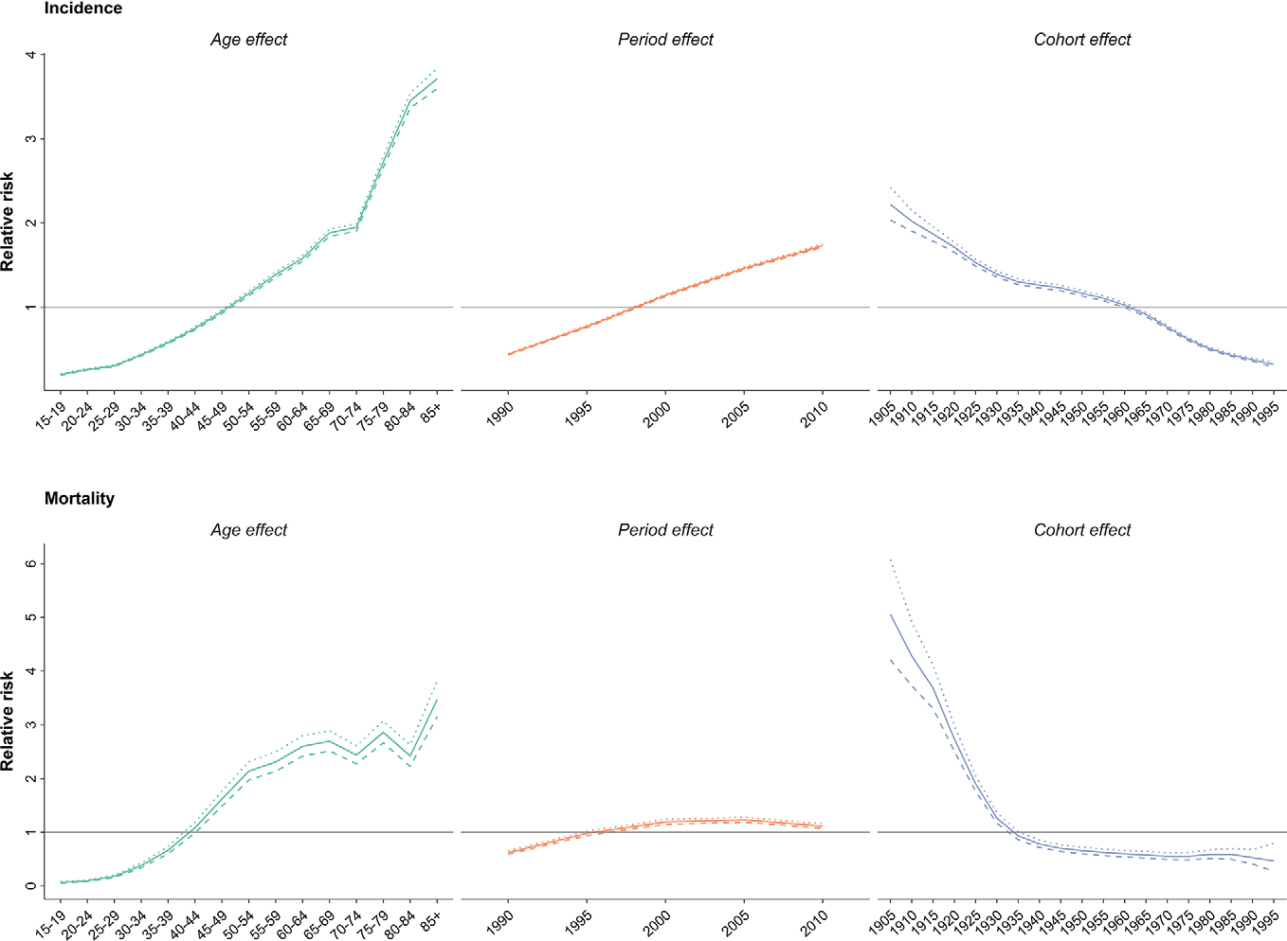
Effect of age, period and cohort on relative risk of incidence (above) and mortality (below); *dotted line: upper limit, dashed line: lower limit*.

Upon evaluating the age-specific incidence, we observed an increasing pattern of incidence rate for all age groups albeit with different incremental slopes. The increment was significantly higher for the age groups of 70–74 years and higher, while the incidence rates for 15–19 years, 20–24 years and 25–29 years of age remained almost steady, with a minimal increase in incidence rates (**Figure 1**). Incidence significantly increased with aging, even after controlling for period and cohort (**Figure 2**). Throughout the study period, the 85+ year-old group had the highest incidence, with an incidence rate of 362.6 (323.5 to 401.3) per 100,000 individuals in 2016, that was 100 times higher than the incidence rate in the 15-19 year-old group with the lowest incidence rates of 3.6 (0.02 to 8.85). Women aged 65–69 years had the highest APC of 5.8%, while for the age-group younger than 35 years APC was less than 3%. A significant increase of breast cancer incidence was observed over the study period. The birth cohort of 1905 had the highest incidence risk, followed by a constant decrease for more recent birth cohorts (**Figure 2**) (**Table S2**).

**Figure 3:**
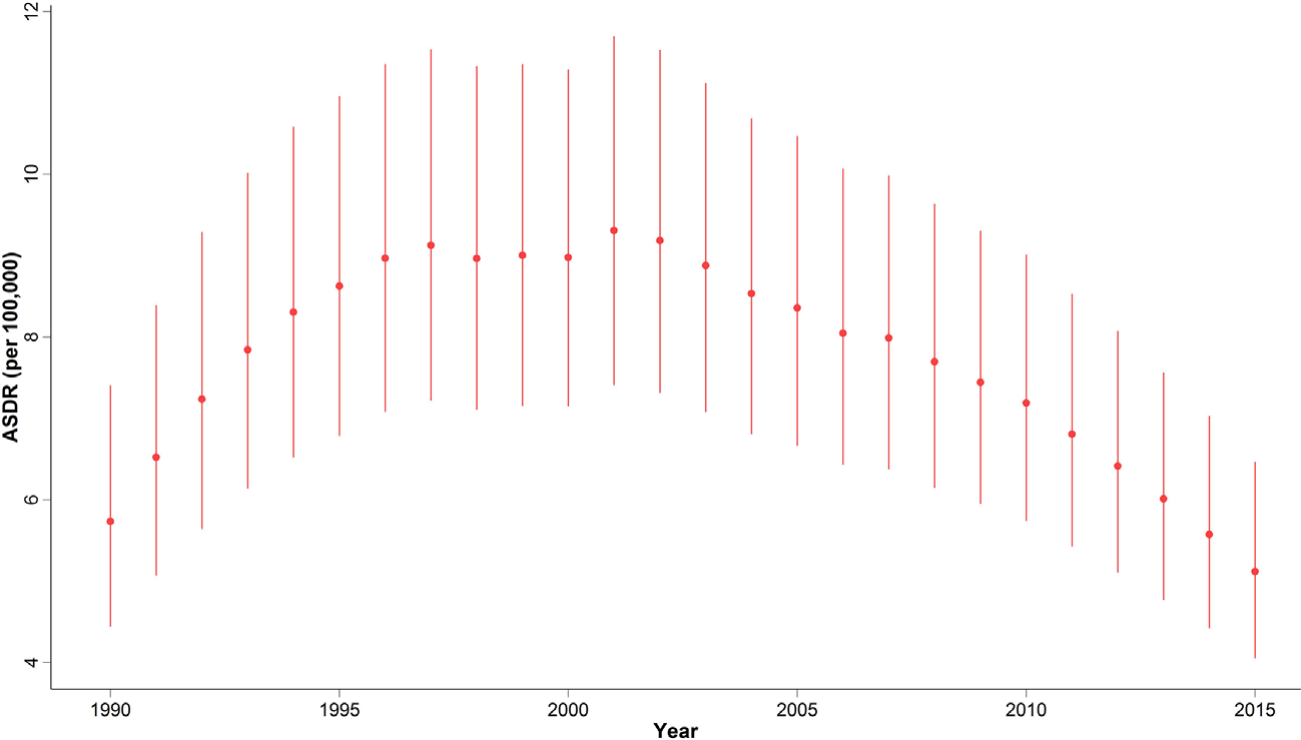
National time trend of ASDR from 1990 to 2015.

In 2015, with 1,608 (1,274 to 2,031) estimated number of deaths, breast cancer accounted for 10% of the total cancer deaths in Iranian women. Despite the overall descending pattern of Age-Standardized Death Rate (ASDR), there was an initial increase of more than 50% from 1990 to 2000, followed by a steady slight decline. ASDR decreased to 5.1 (4.1 to 6.5) deaths per 100,000 individuals in 2015 from 9 (7.1 to 11.3) deaths per 100,000 individuals in 2000, with a negative APC of -0.5% (**Table 1**) (**Figure 3**). Interestingly, despite the negative overall APC, women aged 25–29 and 30–34 had the highest positive APC of 3.6% and 3.1% respectively, while APC was significantly lower for women aged 45–59 years and negative for those older than 60 years of age. The proportion of breast cancer mortality to all-cause mortality in women of reproductive age (15–49 years of age) underwent a significant 4.5-fold increase from 1990. A generally increasing pattern was observed for mortality risk with age, regardless of the time period, with a steeper slope until the age of 50–54 years and minimal fluctuations in the 70+ age groups. From a temporal perspective, after an initial mild increase, a declining mortality risk for all age groups was observed from 2000. Mortality risk significantly declined for birth cohorts of 1905 to 1935 but remained steady for more recent birth cohorts (**Figure 2**).

**Figure 4:**
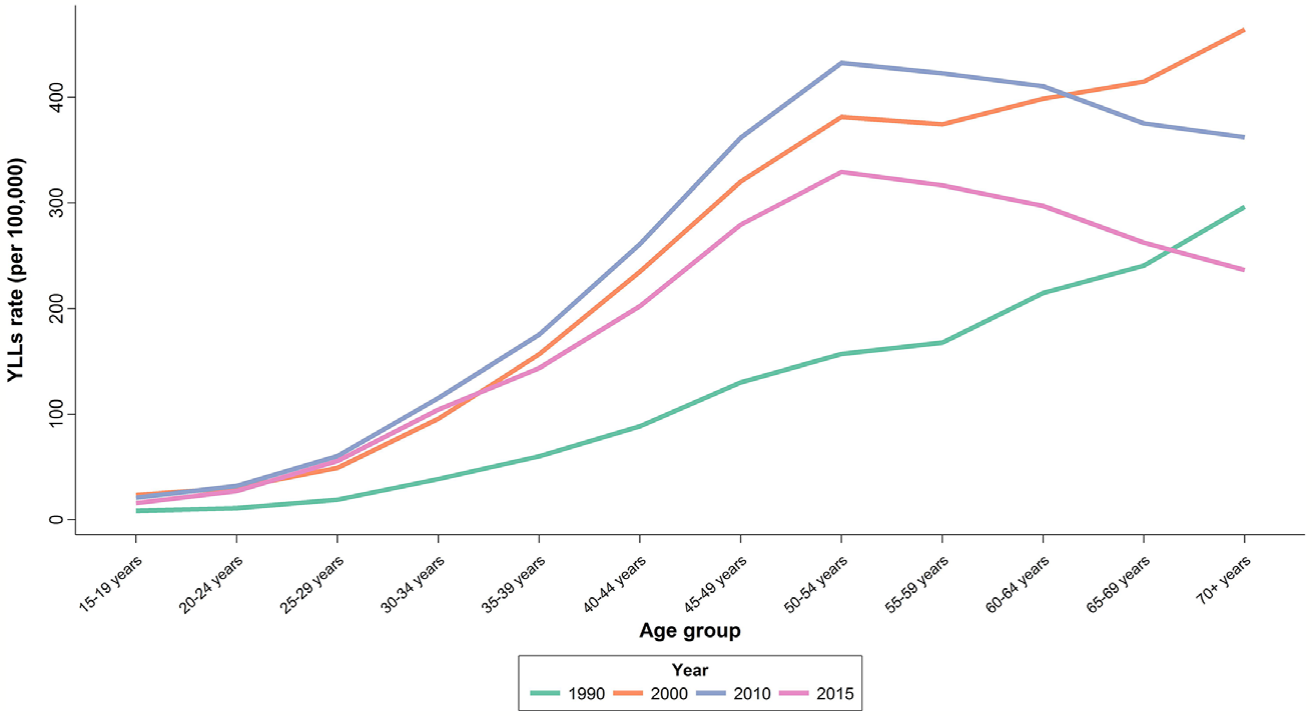
National age trend of YLL in 1990, 2000, 2010, and 2015.

There was an increasing trend in Age-Standardized YLL Rate (ASYR) with an increase from 85.1 (65.6 to 110.4) per 100,000 individuals in 1990 to 196.1 (156.5 to 245.7) per 100,000 individuals in 2010, followed by a slight decline of 20% to 151.1 (119.7 to 190.6) per 100,000 individuals in 2015. APC was calculated at 2.3% for YLL (**Table 1**). In 2015, the highest ASYR was observed in age-groups of 50–54 and 55–59 years with 329.4 (261.7 to 415.5) and 316.6 (249.5 to 400.0) per 100,000 individuals, respectively (**Figure 4**). Similar to what we observed for mortality, APCs of the age groups 25–29 years (4.4%) and 30–34 years (4%) were about twice the overall APC of YLL. Although women aged 35–54 years had lower APCs compared to the aforementioned age groups, their APCs were still higher than the overall calculated percent. APC was negative or significantly lower than the overall percent for those older than 60 years of age.

**Figure 5:**
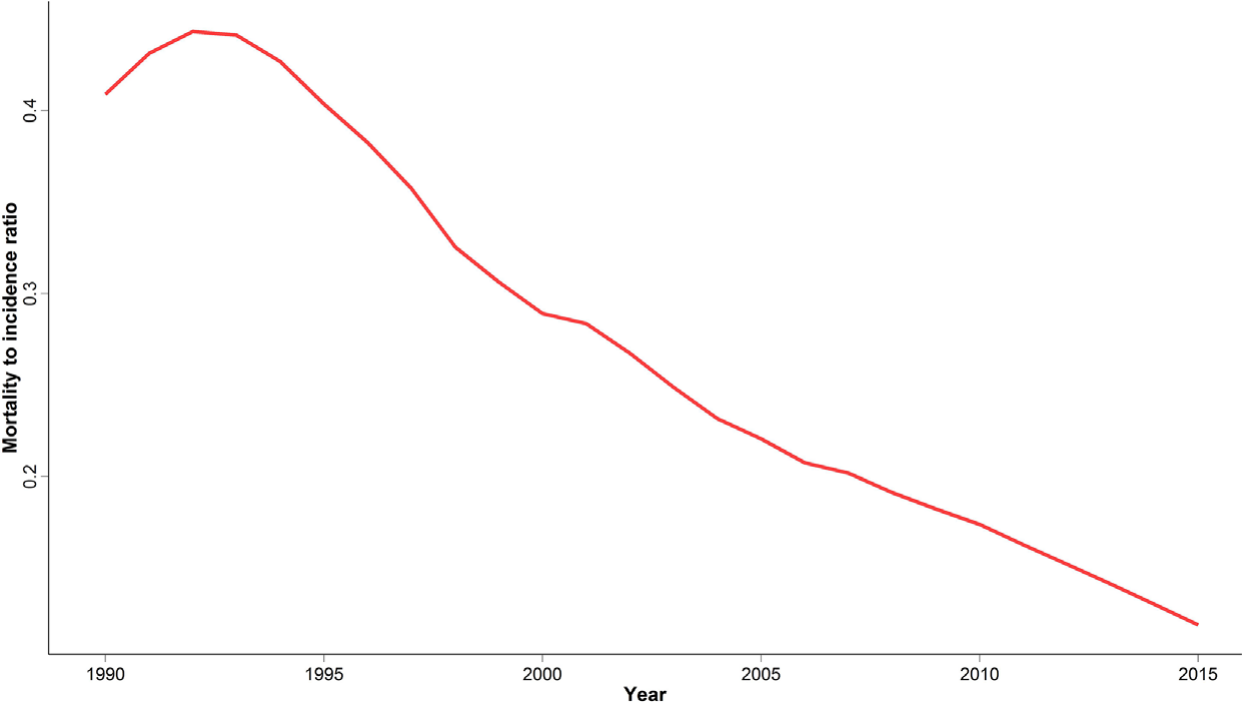
National time trend of mortality to incidence ratio; 1990 to 2015.

We calculated MIR at national and provincial levels to evaluate cancer outcome and quality of care as a potential marker of healthcare provision inequality in different provinces. Despite an initial slight increase of national MIR between 1990 and 1995, we observed a significant four-fold decrease ever since (**Figure 5**). A descending pattern similar to the national MIR was observed for all provinces (**Figure S1**). In addition, the difference between the highest and lowest provincial MIR significantly decreased from 0.58 to 0.19 over the study period.

Based on subnational analysis, the highest ASIR belonged to Tehran, a central province and the capital of Iran, both in 1990 and 2016 with an ASIR of 21.5 (13.3 to 31.4) and 63.7 (53.7 to 74.1) per 100,000 respectively, demonstrating a threefold increase in ASIR similar to national results. In addition to the increasing pattern of ASIR in the majority of provinces, the difference between the provinces with the highest and lowest ASIRs significantly increased by about twofold during the study period (**Figure S2**). Decomposition analysis of the majority of provinces revealed that the greatest attribution to increased incidence belonged to incidence rate change followed by population growth and age structure change, respectively. The highest and lowest increases in the number of new cases belonged to Hormozgan by more than 16 times and Zanjan by about a twofold increase (**Table 1**). A positive APC ranging from 0.8% to 6.8% was calculated for the provincial incidence. A similar increasing trend of incidence was observed in the subnational analysis as well.

The highest ASDR of 2015 was estimated to be 10.0 (7.9 to 12.6) in Alborz, a central province next to Tehran, while in 1990 the highest ASDR belonged to Tehran, at 12.4 (8.9 to 17.1) (**Table 1**). A minor provincial convergence of 20% was observed, mainly attributable to the decline in mortality of provinces with the highest ASDRs. Despite negative APCs in about half of the provinces, women aged 30–34 and 25–29 years had higher and even positive APCs compared to the majority of age groups. Interestingly, we observed this finding among provinces with high and low death rates.

Provinces with the highest and lowest ASYRs were the same as the ASDRs in 1990 and 2016 (**Table 1**). A slight divergence with a 20% increase in difference between provinces with the highest and lowest ASYRs was observed.

## Discussion

In this study, we reported the incidence, mortality and YLL trends of breast cancer in Iran over a 26-year period. A significant constant increase in breast cancer ASIR at national and subnational levels along with expanding provincial disparity was observed. Although mortality has slightly decreased since 2000, a significant positive APC for ASDR was detected in patients aged 25–34 years, leading to raised YLLs in this age group. Despite the higher incidence, decreased mortality has led to a significant declining pattern of MIR and lower provincial MIR disparities.

According to the 2016 GBD study, breast cancer was ranked first among all the cancers in Iran with 11,041 (7844.5 to 12,636.7) new cases in 2016. In addition, an increasing pattern of ASIR with a net increase of 30 per 100,000 individuals since 1990 was reported (4, 34). These findings were highly consistent with our results; albeit, there was a significant difference between death and YLL metrics reported in the GBD and our observations. The trend of breast cancer ASDR in our study increased until 2000, followed by a descending pattern ever since, reaching the minimum of 5.1 per 100,000 individuals in 2015. The GBD reported an ascending trend with a final ASDR value of about twofold (11.6 (9.2 to 12.2) per 100,000 individuals) in 2015, followed by a decline until 2017. Although the trend of ASYR in the GBD was somehow similar to our observations, the reported ASYR in 2015 was two times higher than our reported value, similar to ASDR. A national study from 2006–2010 reported results similar to our findings for all-age death rates, but did not report the ASDR (35). Other studies utilized the GBD or GLOBOCAN data for their modeling and therefore reported higher death rates (36). An explanation for the observed differences can be that our results on mortality were more data-driven than model-driven, using additional data points for the estimation models. Furthermore, we addressed and covered the limitations of the national death registration systems as described in the methods section and the final data were validated by expert physicians in the field of cancer to confirm the accuracy.

Although to our knowledge, there were no published articles covering the period of our study, our findings were consistent with the available literature reporting national and local data over shorter periods. Growing trends of national incidence –overall and for almost all age groups- were reported for 2000–2009 and 2003–2008 and 2014; however, the reported APCs were higher than our calculated APCs (11, 37, 38). Such findings can be due to steeper slopes of the ASIR trend in the timeframe of those studies, which overlap with the initial years of the current study.

Decomposition analysis demonstrated that the effect of age-specific incidence rate was significantly more prominent than population growth and age structure. A part of the rising pattern in incidence, especially in the initial years of the study, is attributable to improvements of the NCR system and increase in the number of patients registered. Improvement of diagnostic tools, more extensive healthcare coverage, raised awareness of the general population on breast cancer symptoms and greater willingness to undergo screening despite cultural barriers and the discomfort of mammography were influential as well. These same factors can explain the rising pattern of the period effect in the age-period-cohort analysis to some extent.

Even though the effectiveness of screening in the early detection of breast cancer has been proven, the extent of participation in screening is not optimal in the Middle East and North African (MENA) region (5, 6, 15). Our findings demonstrated an increased provincial disparity despite the general increasing pattern of incidence in all the provinces. The latter can reflect lower access, knowledge and acceptance toward screening in underprivileged and smaller provinces compared to big cities like Tehran that have multiple diagnostic facilities, cancer specialists, and healthcare coverage in addition to higher educational levels of patients, leading to increased screening participation. The acceptable coverage of screening not only depends on developing well-organized national policies, but also needs interventions to improve knowledge and create a positive attitude toward screening methods in the population at risk (39). A study of the rural areas of Iran demonstrated that the cost-effectiveness of screening programs significantly depended on the extent of participation, highlighting the importance of focusing on improving knowledge and acceptance (40). Almost all MENA countries lack a nationally organized cancer screening program and have numerous cultural and socioeconomic barriers toward screening (5, 41, 42). A review article of breast cancer epidemiology in Iran reported that only about 18% of cases were detected in stage I (43). A recent study in Southern Iran reported stage at diagnosis of II and higher in more than 80% of patients, especially in rural areas [45]. Such findings can be justified by the above-mentioned lack of proper national screening strategies. We recommend establishing national screening and educational policies based on regional risk factors, and cultural and financial settings. Moreover, alterations of the health system toward maximizing access and coverage in the whole country, especially in underprivileged provinces and rural areas seem necessary.

The increase in incidence rate, which was more prominent in big cities, could be a consequence of lifestyle and dietary changes and increased exposure to environmental risk factors. Postmenopausal breast cancer accounted for about 40% of all cancer cases attributable to excess body mass index across the MENA region in 2012 (44). Other potential risk factors in lifestyle including higher fat intake, smoking and low physical activity (especially in post-menopausal women) could be influential in the increased incidence of breast cancer among Iranian women (45–47). Furthermore, altered cultural habits of childbearing such as late age of first pregnancy, nulliparity and using formula milk instead of breastfeeding can also be influential (48–50). However, the definite effect of the abovementioned factors on breast cancer incidence is yet controversial and needs further evaluations. Moreover, accurately defining an influential event on lifestyle and diet patterns similar to effect of the Soviet Union collapse in case of Eastern European countries might not be possible in Iran. Possible justifications in case of Iran can be increasing number of women seeking higher education and increased fulltime employment in early 90s, after Iran-Iraq war that could possibly lead to sedentary lifestyle, more fast food consumption, and difficulties with breastfeeding as a working mother. In addition, higher availability of contraception methods and lower interest in family expansion after a surge in birth rates in late 80s could be influential.

Furthermore, Menopausal Hormone Therapy (MHT) and oral contraceptive pills are widely used nowadays and a number of studies reported an increased breast cancer risk due to increased consumption of these medications to different extents, independent of race and age (51–54). These potential risk factors might have contributed as well to the constant increase of the period effect, as a reflection of lifestyle change in low SDI countries over the study period. To the best of our knowledge, there is no available report on the national trend of attributable risk factors. Even though addressing the correlation between the potential risk factors seems mandatory, it was beyond the scope of the current study. We recommend analyzing trends of risk factors in the general population and registered breast cancer patients at national level to accurately evaluate their magnitude of effect in Iranian women.

Breast cancer had the second highest ASDR among all cancers in the EMRO region and was the leading cause of cancer mortality among women in all countries of the region as well in 2015 (6). The steady but slow declining pattern of mortality and decreased provincial disparity of ASDR since 2000 reflects early detection and improved therapeutic strategies during the study period. However, positive APCs of ASDR and ASYR observed in 25–34 year-old women are alarming. The significant increase in proportion of breast cancer mortality to all-cause mortality in women of reproductive age highlights the importance of urgent assessment of risk factors followed by the design of special diagnostic and treatment policies for this age group. Women younger than 50 years of age had the highest mortality rates in Low Income Countries (LICs) and Low-Middle Income Countries (LMICs) compared to other regions according to the 2012 GLOBOCAN study. Although the incidence of breast cancer is still higher in High-Income Countries (HICs), the burden and YLLs are relatively higher for younger women in LICs & LMICs due to higher mortality rates (55). Furthermore, decomposition analysis in our study demonstrated that less than 25% of increase in number of new cases can be justified by age structure and population growth. A similar study from 1985 to 2005 in Tehran reported similar trend even after age-adjusted analysis (56). Not only breast cancer was most prevalent among Iranian women of 40 to 49 years of age in previous studies, but also about 20% to 30% of cases were younger than 40 years in different studies (43, 56, 57). This finding highlights the importance of more accurately assessing the rise in incidence rate of younger age groups rather than interpreting it solely by younger age structure and higher population of LMICs.

MIR has served as a valuable measure for the long-term evaluation of cancer surveillance and control in earlier studies (58). We observed a significant decline in MIR at national and subnational levels since 1995 in addition to a decrease in provincial MIR disparity. Although the declining MIR generally reflects a combination of better access, improved screening techniques and effective treatment strategies, results should be interpreted with caution. The rapid increase in incidence might be influencing MIR to a higher extent than the slow decline in mortality. Therefore, establishing nation-wide treatment strategies, educational interventions and organized social support of patients for treatment continuum is important in reducing mortality at an acceptable pace and preventing forced migration of patients in underprivileged provinces to big cities to seek proper care. Last but not least, conducting further research on breast cancer epidemiology, with a focus on risk factors and prognostic factors and including them in the NCR would be a proper and cost-effective option for developing practical guidelines and policies. In the next step, proper resource allocation and training human resources for continuous nation-wide policy implementation can lead to early detection, timely treatment and therefore reduce the burden of disease.

We observed an age dependent increase in incidence and mortality as well as temporal increase in incidence and decrease of mortality and a general decline of cohorts in the age-period-cohort analysis. Interestingly, the post-menopausal incidence and mortality pattern in Iran was more similar to western countries such as Germany and US rather than East Asian countries like Japan, South Korea, and China (59–61). These findings can be due to changes in lifestyle and dietary patterns rather than ethnicity in post-menopausal breast cancer. The overall decline of cohort effects for both incidence and mortality was contrary to our expectations, since the prevalence of probable risk factors are increasing in younger generations. Surprisingly, a similar scenario was reported in both western and Eastern Asian countries (59–61). This finding might be due to the cumulative effect of risk factors unknown to us. On the other hand, higher education, improved insight into self-care and fading of cultural taboos might be influential for the younger generations.

To the best of our knowledge, this is the first study reporting the trend of breast cancer incidence, mortality, YLL, MIR, and age-period-cohort at national and subnational levels and with age-period-cohort analysis in Iran over a 26-year period. Furthermore, our findings were more data driven and less dependent on modeling compared to global estimates. Moreover, we have identified and addressed limitations and incompleteness of available registries and tried to minimize the flaws as much as possible.

However, our work had several limitations, including the remaining flaws in the Iranian cancer and death registration systems that can affect the reliability of results as well as limitations in our modeling. Moreover, despite addressing the incompleteness by SSOCR, data from a small proportion of cancer patients not covered by SSI is missing in our model. Furthermore, we used 5-year periods for APC analysis while using shorter periods of 1–2 years can lead to more accurate results. In addition, there were no available registries on screening coverage at national and subnational levels to incorporate in this study for better evaluation of screening efficacy.

## Conclusion

Our findings in this nationally representative study suggest an evident increase of breast cancer incidence in Iran. Further nation-wide evaluation and registration of risk factors and developing and sustaining national screening and prevention policies to tackle both breast cancer and its associated modifiable risk factors, as well as improving awareness on breast cancer across the country is recommended. Current treatment strategies seem to be effective in controlling mortality and reducing YLLs. A descending pattern of MIR at national and subnational levels reflects improved cancer management and quality of care, even though there should be special focus on reducing mortality in younger age groups, since their mortality causes a significant burden on the society.

## Ethics Statement

The ethics committee of the National Institute for Medical Research Development approved the study protocol (IR.NIMAD.REC.1396.192).

## Author Contributions

BA, SN, and FF: Study Concept. FF, NR, and SN: Study Design. FF and NR: Data Acquisition. AH, SM, KG, and EG: Quality Control of Data and Algorithms. AS and KG: Data Analysis and Interpretation. AS, KG, and SM: Statistical Analysis. BA and MS: Manuscript Preparation. SM and YF: Manuscript Editing. FF: Manuscript Review and Supervision. All authors contributed to the article and approved the submitted version.This work was supported by the National Institute for Medical Research Development (Grant number 963348).

## Funding

This work was supported by the National Institute for Medical Research Development (Grant number 963348).

## Conflict of Interest

The authors declare that the research was conducted in the absence of any commercial or financial relationships that could be construed as a potential conflict of interest.

## Supplementary Material

Supplementary Figure 1 Subnational distribution of mortality to incidence ratio in 1990, 1995, 2000, 2005, 2010, and 2015.

Supplementary Figure 2 Subnational time rend of ASIR from 1990 to 2016.
