# Supplementary figures and images for: National and Subnational Incidence, Mortality, and Years of Life Lost Due to Breast Cancer in Iran: Trends and Age-Period-Cohort Analysis Since 1990
Source: Front Oncol. 2021 Mar 25;11:561376. doi: 10.3389/fonc.2021.561376 (PMC8027299; doi:10.3389/fonc.2021.561376)

1990

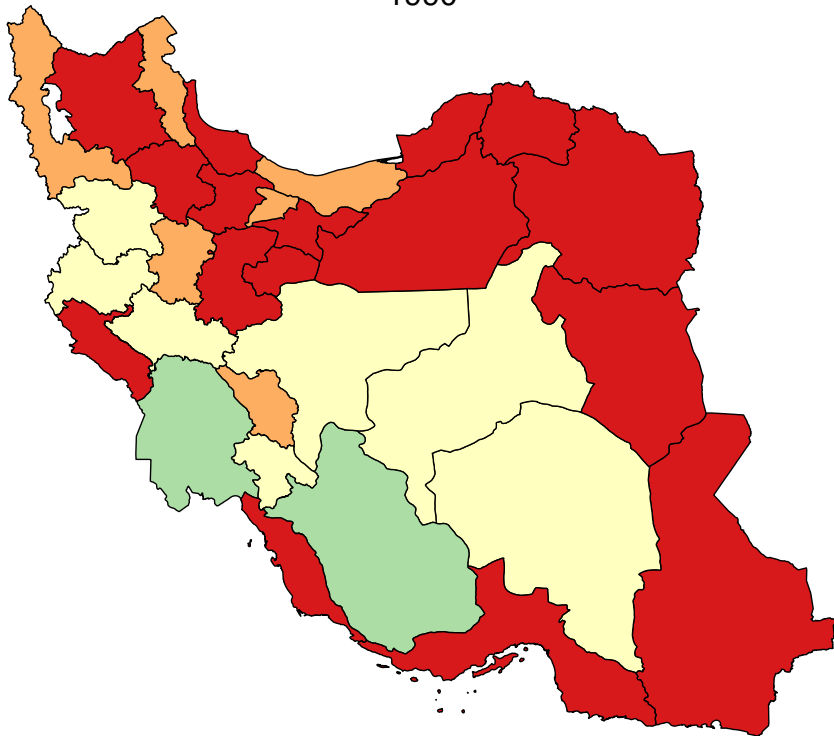

1995

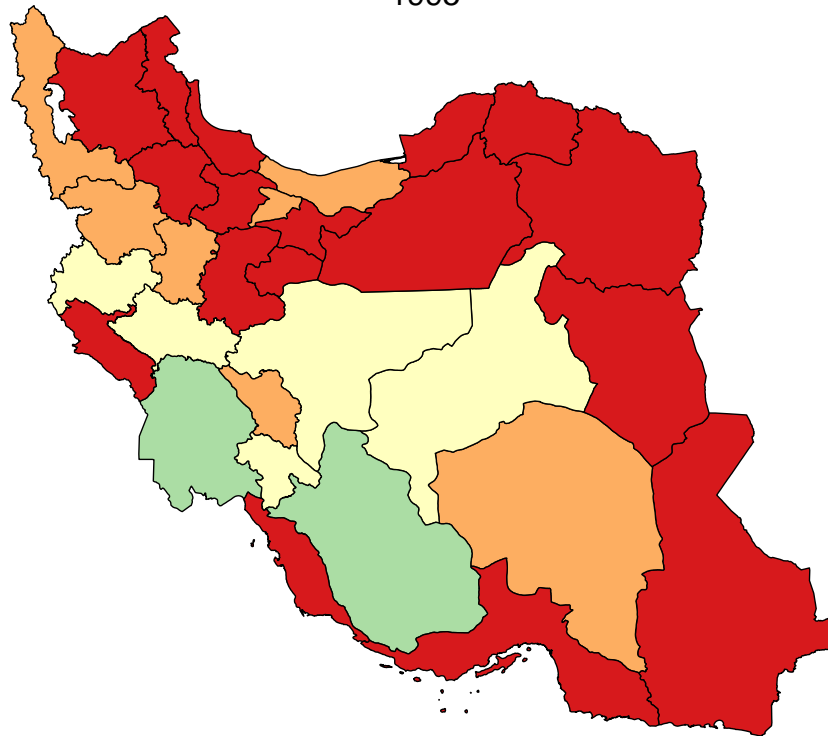

2000

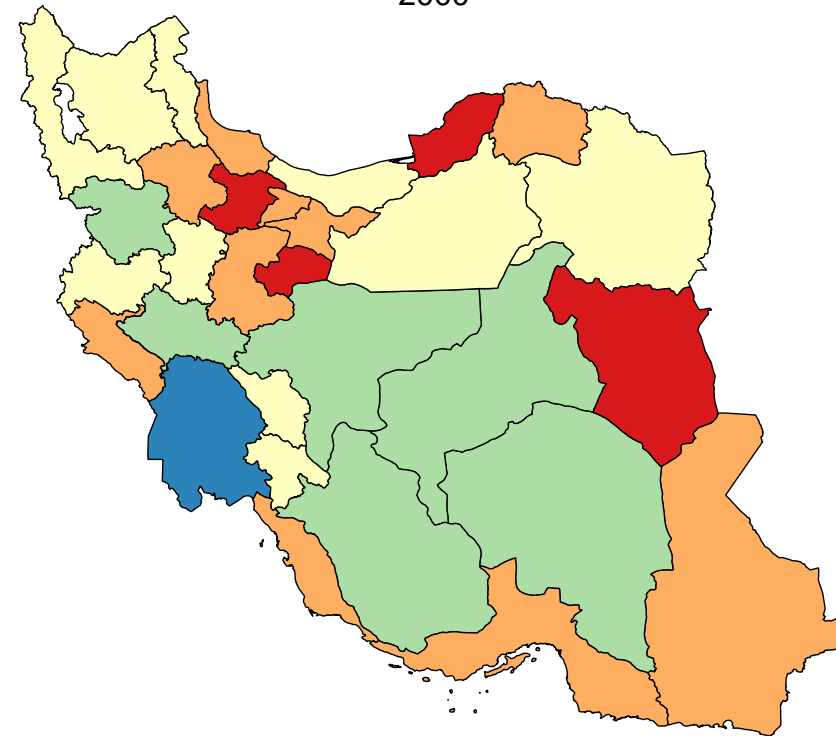

2005

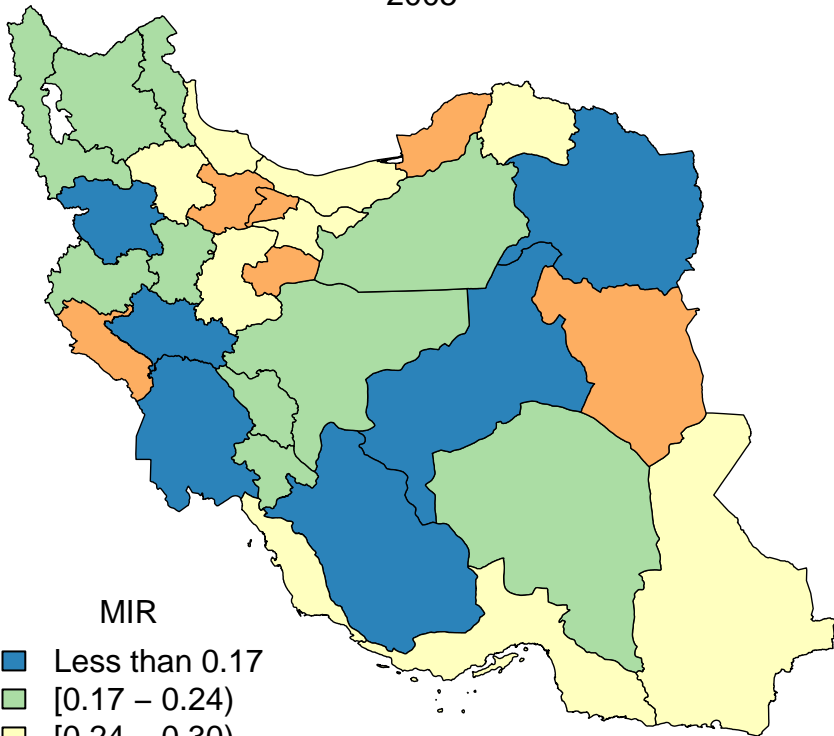

2010

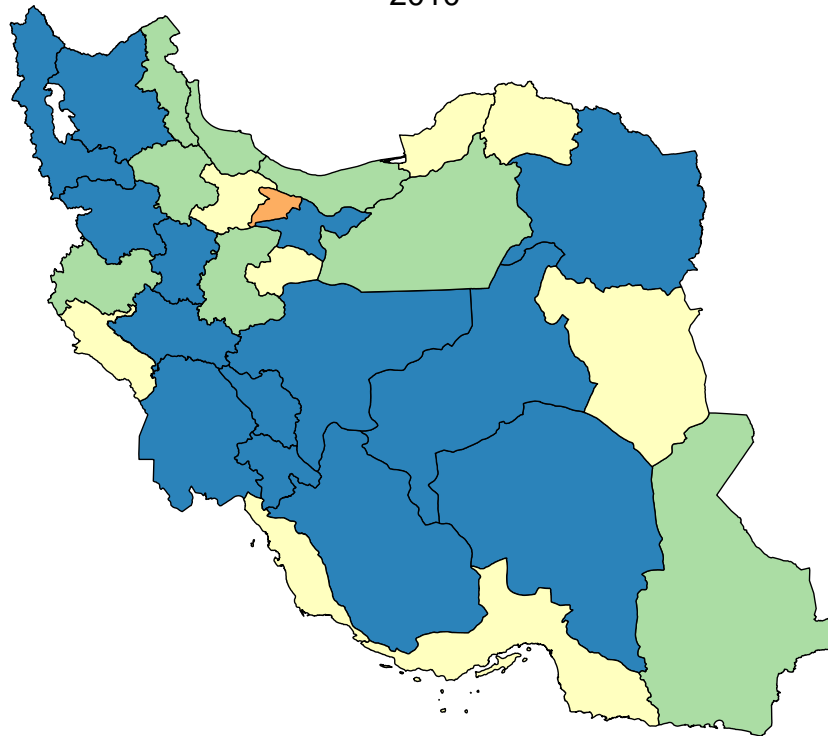

2015

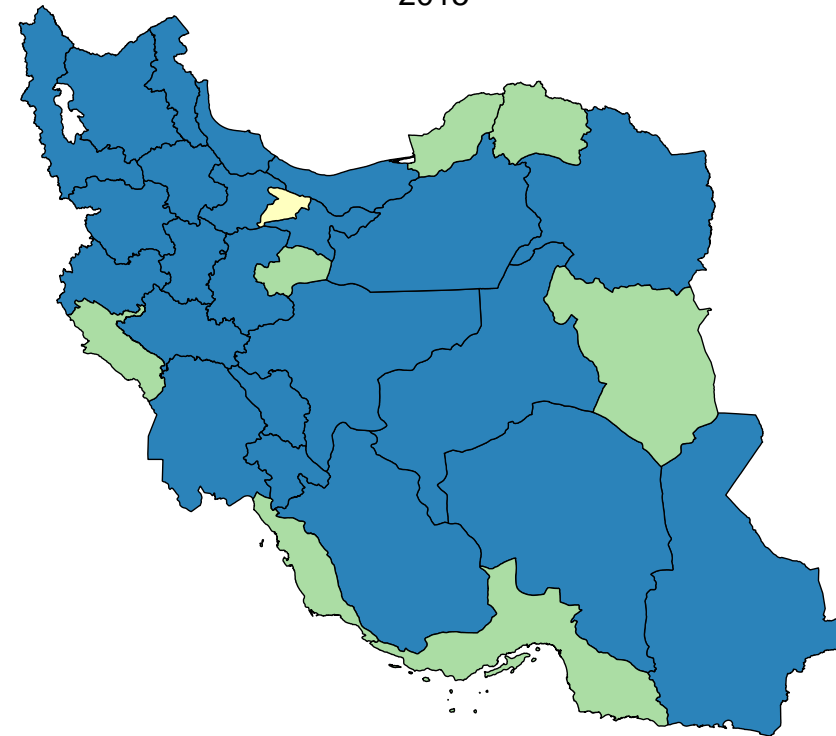

MIR

- Less than 0.17
- [0.17 - 0.24)
- [0.24 - 0.30)
- [0.30 - 0.42)
- More than 0.42

Supplement: Supplementary Figure 1 — Subnational distribution of mortality to incidence ratio in 1990, 1995, 2000, 2005, 2010, and 2015. [file Image_1.pdf]

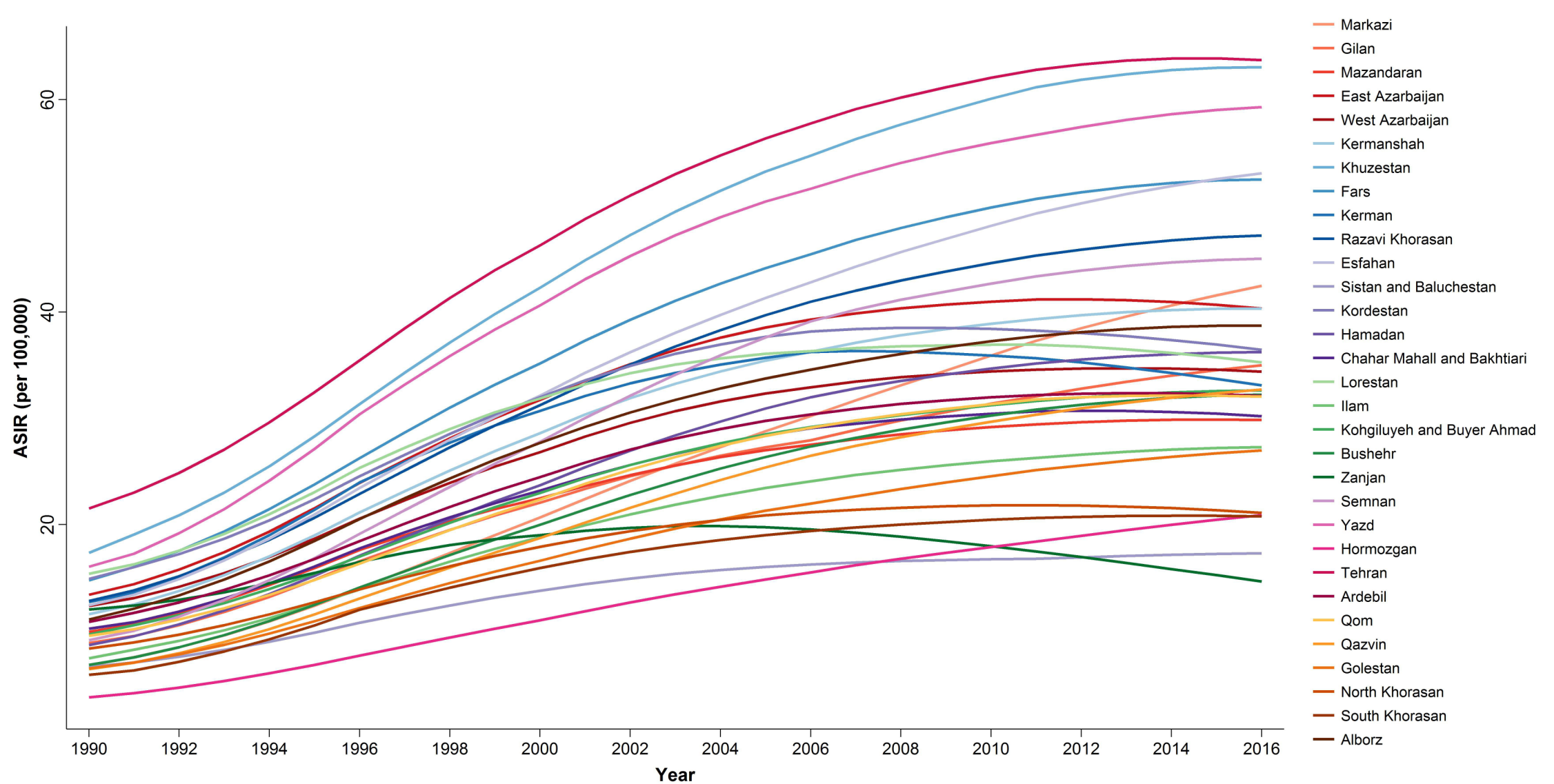

Supplement: Supplementary Figure 2 — Subnational time rend of ASIR from 1990 to 2016. [file Image_2.pdf]
