# Supplementary material for: National and Subnational Incidence, Mortality, and Years of Life Lost Due to Breast Cancer in Iran: Trends and Age-Period-Cohort Analysis Since 1990
Source: Front Oncol. 2021 Mar 25;11:561376. doi: 10.3389/fonc.2021.561376 (PMC8027299; doi:10.3389/fonc.2021.561376)
Supplement: Supplementary file 3 [file Table_1.pdf]

__Table S1__. Incidence decomposition (%) at national and sub-national levels

	Location	New cases		% 1990 - 2016 incident case change cause			Overall change
		1990	2016	Due to population growth	Due to age structure change	Due to incidence rate change	
National		1452	14217	122.4%	67.5%	689.2%	879.0%
Sub-national	Alborz	24	401	279.6%	126.2%	1196.5%	1602.2%
	Ardebil	23	164	80.8%	57.2%	468.7%	606.6%
	Bushehr	9	119	144.5%	49.2%	1063.1%	1256.7%
	Chahar Mahall and Bakhtiari	13	106	105.7%	69.6%	523.9%	699.2%
	East Azarbaijan	92	682	75.2%	69.4%	496.0%	640.6%
	Esfahan	98	1213	117.9%	68.1%	949.4%	1135.5%
	Fars	101	1042	124.9%	65.9%	737.4%	928.1%
	Gilan	48	465	71.9%	68.9%	735.5%	876.3%
	Golestan	15	184	129.0%	59.7%	902.9%	1091.6%
	Hamadan	28	289	78.4%	62.9%	772.6%	913.9%
	Hormozgan	7	116	205.9%	26.6%	1447.6%	1680.1%
	Ilam	6	58	137.5%	53.0%	759.9%	950.4%
	Kerman	45	385	177.3%	57.5%	518.8%	753.6%
	Kermanshah	34	341	103.9%	80.1%	714.5%	898.5%
	Khuzestan	97	1003	154.2%	36.3%	743.1%	933.6%
	Kohgiluyeh and Buyer Ahmad	8	79	150.9%	48.9%	685.8%	885.7%
	Kordestan	35	220	108.7%	50.6%	369.9%	529.2%
	Lorestan	40	251	104.7%	70.0%	350.4%	525.1%
	Markazi	18	281	90.3%	62.7%	1334.6%	1487.5%
	Mazandaran	54	453	93.9%	75.3%	563.7%	732.9%
	North Khorasan	11	73	108.8%	63.2%	407.8%	579.8%
	Qazvin	11	166	120.2%	66.4%	1158.4%	1345.0%
	Qom	14	137	156.0%	50.2%	702.7%	908.9%
	Razavi Khorasan	115	1164	114.1%	64.3%	734.3%	912.7%
	Semnan	10	130	113.4%	41.8%	1027.3%	1182.5%
	Sistan and Baluchestan	16	121	181.4%	32.1%	454.4%	667.9%
	South Khorasan	8	57	55.8%	50.5%	511.7%	617.9%
	Tehran	371	3709	138.0%	97.9%	663.3%	899.2%
	West Azarbaijan	54	442	120.4%	74.9%	530.5%	725.8%
	Yazd	27	301	135.7%	67.3%	829.0%	1031.9%
	Zanjan	21	62	99.1%	51.7%	53.5%	204.3%
