# Supplementary material for: National and Subnational Incidence, Mortality, and Years of Life Lost Due to Breast Cancer in Iran: Trends and Age-Period-Cohort Analysis Since 1990
Source: Front Oncol. 2021 Mar 25;11:561376. doi: 10.3389/fonc.2021.561376 (PMC8027299; doi:10.3389/fonc.2021.561376)
Supplement: Supplementary file 4 [file Table_2.pdf]

Table S2. *Effect of age, period and cohort on incidence and mortality*

Effect	Factor	Incidence			Mortality		
		Coefficient	95% CI	P-Value	Coefficient	95% CI	P-Value
Age	15-19	-1.59	(-1.63 to -1.54)	<0.001	-2.73	(-2.95 to -2.52)	<0.001
	20-24	-1.33	(-1.36 to -1.29)	<0.001	-2.35	(-2.51 to -2.18)	<0.001
	25-29	-1.18	(-1.21 to -1.15)	<0.001	-1.73	(-1.86 to -1.59)	<0.001
	30-34	-0.83	(-0.86 to -0.80)	<0.001	-0.97	(-1.08 to -0.86)	<0.001
	35-39	-0.54	(-0.56 to -0.51)	<0.001	-0.41	(-0.51 to -0.31)	<0.001
	40-44	-0.29	(-0.31 to -0.26)	<0.001	0.08	(-0.01 to 0.17)	0.090
	45-49	-0.05	(-0.07 to -0.03)	<0.001	0.48	(0.40 to 0.57)	<0.001
	50-54	0.15	(0.13 to 0.18)	<0.001	0.76	(0.68 to 0.84)	<0.001
	55-59	0.33	(0.31 to 0.35)	<0.001	0.84	(0.76 to 0.92)	<0.001
	60-64	0.46	(0.44 to 0.48)	<0.001	0.96	(0.88 to 1.03)	<0.001
	65-69	0.63	(0.61 to 0.66)	<0.001	0.99	(0.92 to 1.06)	<0.001
	70-74	0.67	(0.64 to 0.69)	<0.001	0.89	(0.82 to 0.96)	<0.001
	75-79	1.00	(0.98 to 1.03)	<0.001	1.05	(0.98 to 1.12)	<0.001
	80-84	1.24	(1.21 to 1.26)	<0.001	0.89	(0.80 to 0.97)	<0.001
	85+	1.31	(1.28 to 1.34)	<0.001	1.24	(1.15 to 1.34)	<0.001
Period	1990-1994	-0.81	(-0.83 to -0.79)	<0.001	-0.47	(-0.53 to -0.42)	<0.001
	1995-1999	-0.26	(-0.27 to -0.24)	<0.001	-0.02	(-0.06 to 0.02)	0.306
	2000-2004	0.14	(0.12 to 0.15)	<0.001	0.18	(0.14 to 0.21)	<0.001
	2005-2009	0.38	(0.37 to 0.39)	<0.001	0.21	(0.17 to 0.25)	<0.001
	2010-2014	0.55	(0.54 to 0.56)	<0.001	0.11	(0.07 to 0.15)	<0.001
Cohort	1905	0.80	(0.71 to 0.89)	<0.001	1.62	(1.44 to 1.80)	<0.001
	1910	0.70	(0.64 to 0.77)	<0.001	1.45	(1.32 to 1.59)	<0.001
	1915	0.63	(0.58 to 0.67)	<0.001	1.30	(1.20 to 1.41)	<0.001
	1920	0.54	(0.50 to 0.57)	<0.001	1.01	(0.92 to 1.09)	<0.001
	1925	0.43	(0.40 to 0.45)	<0.001	0.64	(0.57 to 0.72)	<0.001
	1930	0.33	(0.31 to 0.36)	<0.001	0.23	(0.16 to 0.31)	<0.001
	1935	0.27	(0.24 to 0.29)	<0.001	-0.07	(-0.15 to 0.01)	0.104
	1940	0.24	(0.21 to 0.26)	<0.001	-0.24	(-0.33 to -0.16)	<0.001
	1945	0.21	(0.18 to 0.24)	<0.001	-0.36	(-0.45 to -0.27)	<0.001
	1950	0.16	(0.13 to 0.18)	<0.001	-0.42	(-0.51 to -0.32)	<0.001
	1955	0.10	(0.08 to 0.13)	<0.001	-0.47	(-0.57 to -0.37)	<0.001
	1960	0.03	(0.00 to 0.05)	0.039	-0.51	(-0.61 to -0.41)	<0.001
	1965	-0.09	(-0.11 to -0.06)	<0.001	-0.55	(-0.66 to -0.44)	<0.001
	1970	-0.26	(-0.29 to -0.24)	<0.001	-0.59	(-0.71 to -0.48)	<0.001
	1975	-0.48	(-0.51 to -0.45)	<0.001	-0.59	(-0.72 to -0.47)	<0.001
	1980	-0.67	(-0.70 to -0.64)	<0.001	-0.53	(-0.67 to -0.40)	<0.001
	1985	-0.83	(-0.87 to -0.79)	<0.001	-0.53	(-0.70 to -0.36)	<0.001
	1990	-0.97	(-1.02 to -0.92)	<0.001	-0.64	(-0.89 to -0.38)	<0.001
	1995	-1.13	(-1.22 to -1.03)	<0.001	-0.76	(-1.29 to -0.23)	0.005
Constant		-7.99	(-8.00 to -7.98)	<0.001	-9.34	(-9.38 to -9.30)	<0.001
Deviance		49.51			118.78		
AIC		10.83			10.26		
BIC		-118.88			-49.60		

CI, Confidence Interval; AIC, Akaike Information Criterion; BIC, Bayesian Information Criterion
